# Impaired processing of threat in psychopathy: A systematic review and meta-analysis of factorial data in male offender populations

**DOI:** 10.1371/journal.pone.0224455

**Published:** 2019-10-29

**Authors:** Petya Kozhuharova, Hannah Dickson, John Tully, Nigel Blackwood

**Affiliations:** 1 Department of Forensic and Neurodevelopmental Sciences, Institute of Psychiatry, Psychology and Neuroscience, King’s College London, London, United Kingdom; 2 Centre for Cognition, Neuroscience and Neuroimaging (CNNI), Department of Psychology, Roehampton University, London, United Kingdom; Victoria University of Wellington, NEW ZEALAND

## Abstract

**Background:**

Psychopathy is a personality disorder characterised by two underlying factors. Factor 1 (affective and interpersonal deficits) captures affective deficits, whilst Factor 2 (antisocial and impulsive/disorganised behaviours) captures life course persistent antisocial behaviours. Impaired processing of threat has been proposed as an aetiologically salient factor in the development of psychopathy, but the relationship of this impairment to the factorial structure of the disorder in adult male offenders is unclear.

**Objectives:**

To investigate whether threat processing deficits are characteristic of psychopathy as a unitary construct or whether such deficits are specifically linked to higher scores on individual factors.

**Data sources:**

A systematic review of the literature was conducted by searching PubMed, Web of Science and PsycINFO.

**Methods:**

Studies were included if they (1) reported physiological measures of threat response as the primary outcome measure (2) indexed psychopathy using a well-validated clinician rated instrument such as the PCL-R (3) investigated male offenders between 18 and 60 years of age (4) reported threat processing analyses using both Factor 1 and Factor 2 scores (5) provided sufficient data to calculate effect sizes and (6) were published in English-language peer-reviewed journals. We identified twelve studies with data on 1112 participants for the meta-analysis of the relationship with Factor 1 scores, and nine studies with data on 801 participants for the meta-analysis of the relationship with Factor 2 scores. We conducted the meta-analyses to calculate correlations using random-effects models.

**Results:**

PCL-R/SV Factor 1 scores were significantly and negatively related to threat processing indices (r = -0.22, (95%CI [-0.28, -.017]). Neither PCL-R/SV Factor 2 scores (r = -0.005, 95%CI [-0.10, 0.09]), nor PCL-R total score (r = -0.05, (95%CI [-0.15, -0.04]) were related to threat processing indices. No significant heterogeneity was detected for the Factor score results.

**Conclusions:**

The meta-analyses of the distinct psychopathy factors suggest that the threat processing deficits observed in male offenders with psychopathy are significantly associated with higher scores on Factor 1. A similar relationship does not exist with Factor 2 scores. Our findings highlight the importance of investigating the potentially discrete relationships between aetiological variables and the two factor constructs in the disorder.

## Introduction

Violence is a global public health problem, with most violent crimes being committed by a small group of males who meet diagnostic criteria for conduct disorder in childhood and for antisocial personality disorder (ASPD) in adulthood [1]. Within this population, a subgroup of individuals additionally presents with psychopathy. This is a severe personality disorder encompassing two distinguishable symptomatic factors–affective and interpersonal deficits (interpersonal manipulation, callousness, shallow affect, lack of empathy, known as Factor 1 traits) and life course persistent antisocial and impulsive behaviours (impulsive and reckless behaviour, juvenile delinquency, and early behavioural problems, known as Factor 2 traits) [2]. The antisocial personality disordered group with additional diagnoses of psychopathy begin offending at a younger age, commit a disproportionate number of violent offences, typically fail to benefit from rehabilitation programs and present with higher rates of violent recidivism on release from custodial settings [3].

One measure that has been identified as potentially aetiologically salient in the psychopathic group is the aberrant processing of threatening cues in the social environment [4]. Threat processing is defined as the automatic bodily reactivity to threatening stimuli which elicits defensive responses [5]. Threat processing therefore denotes the activation of a neurobiological mechanism which prepares an organism to react appropriately to imminent threat. In healthy individuals, presentation of aversive or threatening cues such as a shock or loud noise in conditioning paradigms, or startle probes while viewing unpleasant pictures, results in the mobilization of defensive actions, which can be measured by threat-associated responses such as skin conductance levels and startle reflex responding [5, 6, 7]. These autonomic and central nervous system responses are hypothesised to reflect responses to the dimensional aspects of such threatening cues, namely arousal and valence [8, 9], and underpin both the core affective response to such cues, and the preparation for instrumental action [10, 11].

Many studies have demonstrated an abnormal response to aversive stimuli in antisocial individuals, particularly those with high psychopathic traits. For example, Lykken’s landmark study [12] showed that psychopathic individuals had diminished skin-conductance reactivity to a conditioned stimulus associated with shock and less avoidance of punished responses on an avoidance learning task. These findings gave rise to the low-fear hypothesis of psychopathy, positing threat processing deficits as the core underlying feature of the disorder [12]. Numerous studies have since provided support for this theory by demonstrating that offenders with high psychopathic traits show smaller electrodermal responses when anticipating aversive shock [13–17]. Psychopathic individuals also show reduced autonomic reactivity relative to non-psychopathic individuals while processing unpleasant visual images capable of provoking a distressed or fearful response, as expressed by diminished or absent startle modulation and skin-conductance responses [18–22]. Further, startle potentiation in response to aversive events [23, 24] and anticipatory skin conductance response [25] are known to be mediated by a “limbic” network including vmPFC, the amygdala, the thalamus and brainstem (including the peri-aqueductal grey [PAG] and locus coeruleus), suggesting a functional deficit in the amygdala or affiliated structures in psychopathic individuals. Consistent with this, neuroimaging studies of psychopathic individuals have suggested that impaired amygdalar activation occurs during threat processing paradigms including fear conditioning and instrumental learning tasks [26–31].

Recent studies have suggested that deficits in threat processing, such as abnormal responding to aversive stimuli, are more characteristic of Factor 1 of the psychopathy construct (affective and interpersonal deficits). Factor 2 (antisocial and impulsive/disorganised behaviours) scores appear more related to impaired cognitive-executive functioning [32]. In keeping with this, investigations of the physiological measures of threat processing, such as fear-potentiated startle responses and startle blink modulation during aversive stimulation, have shown reduced reactivity in individuals scoring high on Factor 1, but not on Factor 2 [22, 33, 34]. Similarly, reduced skin-conductance response during anticipation of aversive stimuli, one of the most replicated findings in psychopathic individuals, has recently been distinctively associated with Factor 1 [35].

Taken together, these studies suggest that the impaired threat processing seen in psychopathy may be particularly related to Factor 1 (affective and interpersonal deficits) scores in this group. Negatively valenced stimuli do not elicit the same defensive response as they do in non-psychopathic antisocial populations and healthy controls. Further support for this conclusion comes from recent findings indicating that controlling for the correlation between Factor 1 and Factor 2 strengthens the negative association between Factor 1 and threat processing, whilst having no effect on the association between Factor 2 and threat processing [36–38]. Using a global measure of psychopathy based on combined Factor 1 and Factor 2 scores provides limited insights when considering the underlying aetiology of the social cognitive abnormalities in the disorder. A meta-analysis examining the processing of facial or vocal emotional information in psychopathy [39], demonstrated that while the unitary construct of psychopathy was found to be associated with pervasive emotion recognition deficits, a targeted analysis showed that Factor 1 scores were only related to deficits in recognising fear, while Factor 2 scores were associated with deficits in recognising other emotions [39].

Threat processing and other aetiological components of psychopathy may therefore also be best understood and investigated as being related in different ways to Factor 1 and Factor 2 traits within the disorder. To date however, no systematic review or meta-analysis has attempted to disentangle the link between the factorial constructs of psychopathy and threat processing impairments. Consequently, it remains unclear whether the observed deficits in threat processing are characteristic of the condition or of only one of its constituent factors. This ambiguity needs to be resolved to help to promote a better understanding of causal mechanisms and to help to develop effective interventions [40]. To our knowledge, only one previous systematic review investigating threat processing in psychopathy (dimensionally conceptualised to include clinician-assessed offender samples and self-rated community and student populations) has been published [4]. The review aimed to determine whether the fear processing abnormalities in psychopathy were best characterised as impairments in automatic threat processing, impairments in the conscious experience of fear, or both. The findings suggested that psychopathy is characterised by impaired automatic threat processing. However, their analysis of the relationship between the distinct psychopathy factors and threat processing returned nonsignificant results. The current work will seek to extend these findings by examining automatic threat processing in psychopathy, but solely in the context of offender populations subject to detailed clinician assessment in studies that report factor-based analyses. Furthermore, the project uses standardised PRISMA approaches to reporting to ensure clarity and transparency of the review process [41]. Research has suggested that community samples manifest lower degrees of both psychopathy factors and predominantly possess the affective deficits with relatively reduced degrees of antisocial features (whereas offenders with psychopathy possess high scores on both factors [42, 43]). The strength of the association between the two factors is also stronger among offender in comparison to community samples [44]. Restricting our consideration to offender populations therefore serves to limit confounds and to ensure consistency across included studies. The aim of the present work was to systematically review the psychopathy literature which has reported factorial data and conduct meta-analyses to examine whether threat processing deficits are characteristic of psychopathy as a unitary construct or whether such deficits are specifically linked to higher scores on individual factors. Based on findings in previous work, we hypothesised that impaired threat processing would be related to higher scores on Factor 1 items of the disorder.

## Methods

The systematic review and meta-analyses were conducted following the Preferred Reporting Items for Systematic Reviews and Meta-Analyses [41] guideline.

### Search strategy

We searched for studies indexed in three databases from their start dates: PsycINFO (1960–28 February 2019), PUBMED (1960–28 February 2019) and Web of Science (1945–28 February 2019). Combinations of search terms relating to threat processing (threat OR fear OR arousal) and psychopathy (psychopathy OR psychopathic OR antisocial OR “offender sample” OR “forensic sample” OR “antisocial personality”) were used. On PsycINFO, additional limits were used for the methodology (male population groups) and publication type (peer reviewed); the other databases did not provide the function required to enable these limits. Reference lists were scanned by hand to identify additional studies. Non-English language articles were excluded.

To ensure rigorous systematic search and identification of all relevant papers, we carried out an additional systematic search looking for studies utilising neuroimaging metrics of threat responsivity. The same databases were searched with a combination of the following search terms: (fear OR threat OR arousal) AND (functional imaging OR functional MRI or fMRI) AND (psychopathy OR psychopathic OR antisocial OR ''offender sample'' OR ''forensic sample'' OR ''antisocial personality''). This secondary search did not reveal any additional papers.

### Study eligibility

Threat processing studies had to report physiological measures of threat response as the primary outcome measure (i.e. the dependent variable in analyses). These physiological indices of autonomic nervous system activation included skin conductance response, heart rate, blood pressure, startle blink reflex, fear potentiated startle, theta coherence, event related potentials or neuroimaging derived metrics [6]. Psychopathy had to be defined using a well-validated clinician administered instrument (the PCL-R [2] or SV [45] instrument). Studies were included if a) they investigated male offenders between the ages of 18 and 60 with current or historical criminal convictions, b) they employed sample sizes greater than 10 participants (following guidance on required sample size for accurate effect size estimation, [46]), c) they reported threat processing analyses using factor-based approaches (that is, their analytic approach enabled factor level data to be appraised) d) they provided sufficient data to calculate effect sizes for the separate factor analyses and e) they were published in English-language peer-reviewed journals.

Studies were excluded if a) they examined only female offenders (because psychopathy may be differentially expressed across biological sex [47, 48]), and if b) they had included participants with brain injuries, learning disabilities or major mental illnesses such as schizophrenia or bipolar affective disorder. When suitability for inclusion was in question, this was resolved through discussion between the authors. No effects from non-published data were included in this analysis.

**Fig 1:**
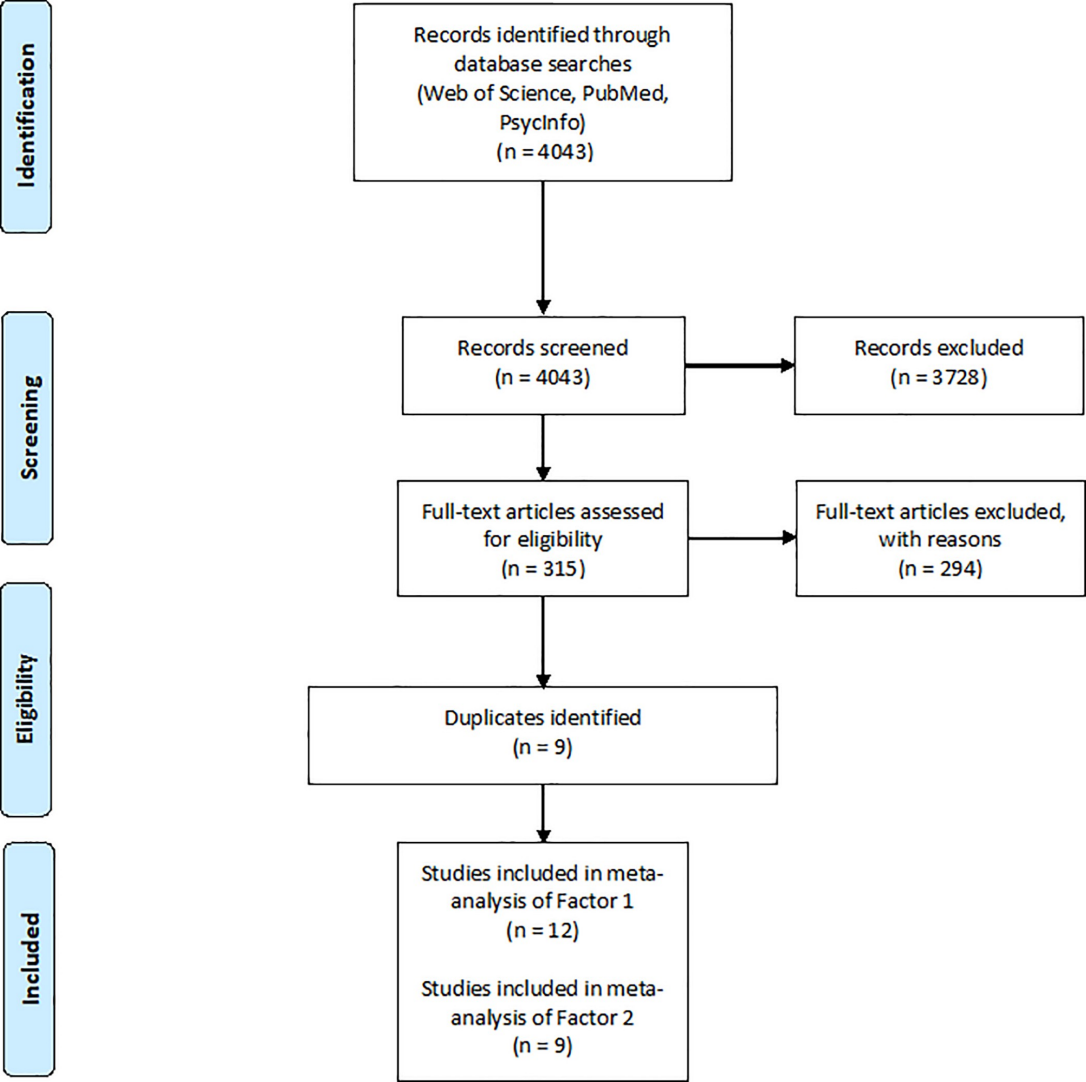
Flowchart of the systematic search strategy.

Twelve studies involving 1112 participants were included in the meta-analysis of the relationship between threat processing indices and Factor 1 scores. Nine studies involving 801 participants were included in the meta-analysis of the relationship between threat processing indices and Factor 2 scores. This is due to some papers not providing specific effect sizes for Factor 2 (instead, choosing solely to report the relevant results as ‘‘non-significant”). Fig 1 illustrates the paper selection process (see S1 Table in supplementary material for details on number of papers and reasons for exclusions).

### Data extraction

A standardized form was used to extract data based on a template by the Cochrane Consumers and Communication Review Group (2016) and refined for the purposes of the current paper in view of the use of cross-sectional studies. The following information was collected: (1) authors and year of publication, (2) methods and measures (i.e. tasks), (3) sample size, (4) psychopathy assessment instrument, (5) physiological index of threat processing and (6) main findings. Studies did not report data from overlapping samples.

### Quality assessment

To ascertain the quality and susceptibility to bias of individual studies the authors tailored a ten-item scale using items from the STROBE Statement for cross-sectional studies (see S1 File, [49]). Each item was scored 0 or 1. The total score range was 0 to 10. The quality index was calculated at the study level by summing the items across all criteria. Uncertainties about quality were resolved through discussions between authors. Samples were considered of low quality if they scored from 0 to 3 points; medium quality, from 4 to 6 points; and high quality, from 7 to 10 points.

### Statistical analysis

All analyses were completed using the meta package for R [50]. The meta-analyses were performed using a random effects model, as we expected considerable heterogeneity due to the small number of studies [51]. Pearson’s r was used as a measure of effect size and was transformed to Fisher’s z for the purposes of analyses [52]. The pooled effect size and its confidence intervals were converted back into the original scale and reported as such. Standardized beta coefficients were converted to r’s using the procedures outlined by Peterson [53]; relevant F value statistics were converted to r using formulas outlined by Field [54]. The relevant beta and F statistics were taken from models including other predictors: S2 Table provides a summary of these models. Cohen’s [55] rules for interpretation were used: r ~ 0.10 is a small effect size, r ~ 0.30 is a medium effect size, r ~ 0.50 is a large effect size.

We tested for heterogeneity with the chi-squared test *Cochran’s Q and I^2^ statistics* [56]. The heterogeneity analyses were performed with a random-effects model, with 95% confidence intervals and a two-tailed test. If heterogeneity tests returned significant results, we planned to conduct a further moderator analysis via meta-regression with quality of studies as a moderator (low/moderate/high).

Potential publication bias for relationships with factor 1 and factor 2 scores were assessed graphically and statistically using published methods [57–59].

A summary of the characteristics of the eligible studies and their respective quality indices is included in Table 1. Three studies were classified as having lower quality, six as intermediate and three as higher quality studies.

**Table 1:** Characteristics of studies included in the meta-analyses.

*Study [ref]*	*Methods and measures*	*Participants*	*Psychopathy* *Measure*	*Outcome*	*Main findings* *Factor 1*	*Main findings* *Factor 2*	*Quality index*
Newman et al, 2010 *‡ [14]	Fear conditioning paradigm	125 offenders	PCL-R	Fear-potentiated startle (FPS)	Factor 1 was negatively and significantly associated with outcome.	No data on Factor 2.	4
*Vaidyanathan et al, 2011 [33]*	Startle modulation during affective picture-viewing task	108 offenders	PCL-R	Startle potentiation	Factor 1 was negatively and significantly associated with outcome.	Factor 2 was negatively and not significantly associated with outcome.	6
*Veit et al, 2013 [60]*	Fear conditioning paradigm	14 offenders	PCL-R	Skin Conductance Response (SCR)	Factor 1 was negatively and not significantly associated with outcome.	Factor 2 was negatively and not significantly associated with outcome.	4
*Baskin-Sommers et al, 2013 ‡ [61]*	Startle modulation during affective picture-viewing task	136 offenders	PCL-R	Emotion modulated startle	Factor 1 was negatively and significantly associated with outcome.	Factor 2 was not associated with outcome.	5
*Venables, 2015 ‡ [32]*	Aversive noise during affective picture-viewing task	139 offenders	PCL-R	Late positive potential (LPP, measure of affective processing)	Factor 1 was negatively and significantly associated with outcome.	Factor 2 was positively and not significantly associated with outcome.	7
*Drislane et al, 2013 [62]*	Noise probes during affective picture-viewing task	140 offenders	PCL-R	Event related potentials	Factor 1 was negatively and significantly associated with outcome.	Factor 2 was positively and not significantly associated with outcome.	4
Baskin-Sommers et al, 2011a *‡ [63]	Fear conditioning paradigm	87 offenders	PCL-R	Fear-potentiated startle (FPS)	Factor 1 was negatively and significantly associated with outcome.	No data on Factor 2.	6
*Sadeh & Verona, 2012 [64]*	Startle probe during an affective-picture viewing task	63 offenders	PCL-SV	Fear-potentiated startle (FPS)	Factor 1 was negatively and not significantly associated with outcome.	Factor 2 was positively and not significantly associated with outcome.	6
*Casey et al., 2013 † [65]*	Emotion regulation during affective picture-viewing task	61 offenders	PCL-R	Cardiovascular response (heart rate)	Factor 1 was negatively and significantly associated with outcome.	Factor 2 was not associated with outcome.	6
*Verona et al., 2012 [66]*	Emotional processing in an emotional-linguistic Go/No-Go task	45 offenders	PCL-SV	P3 event related potentials	Factor 1 was negatively and not significantly associated with outcome.	Factor 2 was positively and significantly associated with outcome.	7
*Baskin-Sommers et al., 2011b ‡ [67]*	Fear conditioning paradigm	92 offenders	PCL-R	Fear-potentiated startle (FPS)	Factor 1 was negatively and significantly associated with outcome.	Factor 2 was negatively and not significantly associated with outcome	8
Tillem et al., 2016 *‡ [68]	Picture-viewing paradigm (threat vs neutral pictures)	99 offenders	PCL-R	EEG theta-coherence	Factor 1 was negatively and significantly associated with outcome.	No data on Factor 2.	5

* Only included in the meta-analysis of Factor 1. This is due to specific papers not providing enough information to calculate effect sizes for Factor 2 (stated as non-significant in the papers).

† Reported standardized beta coefficients, which were converted to r’s

‡ Reported relevant F value statistics, which were converted to r’s

## Results

To test whether threat processing is associated with psychopathy as a unitary construct, we carried out pooled analysis of the total PCL-R scores and threat processing measures. The total psychopathy score was not significantly associated with threat processing metrics, r = -0.05 (95% CI [-0.15, - 0.04]). Significant heterogeneity was detected across the pooled studies (Q2 = 20.70, df = 11, p = 0.04/ I^2^ = 46.9%), indicating that there is considerable variation in study outcomes between the included studies (see S1 Fig). Visual inspection of the funnel plot did not suggest presence of publication bias (see S2 Fig).

### Factor 1

**Fig 2:**
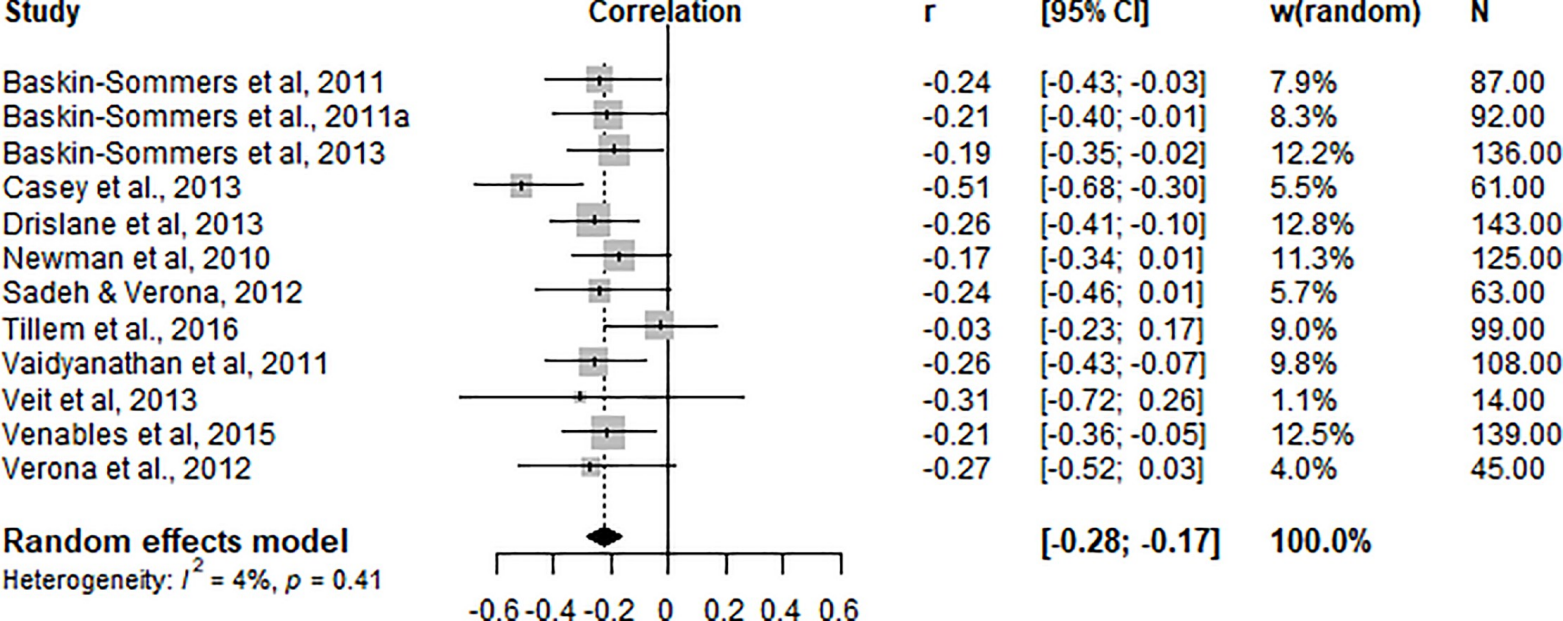
Correlations (r) between physiological threat processing index and PCL-R/SV Factor 1 scores.

As shown in Fig 2, the pooled analysis of 12 studies showed that Factor 1 (affective and interpersonal deficits) scores had a negative and significant moderate effect on threat processing indices, r = -0.22 (95% CI [-0.28, -0.17]).

**Fig 3:**
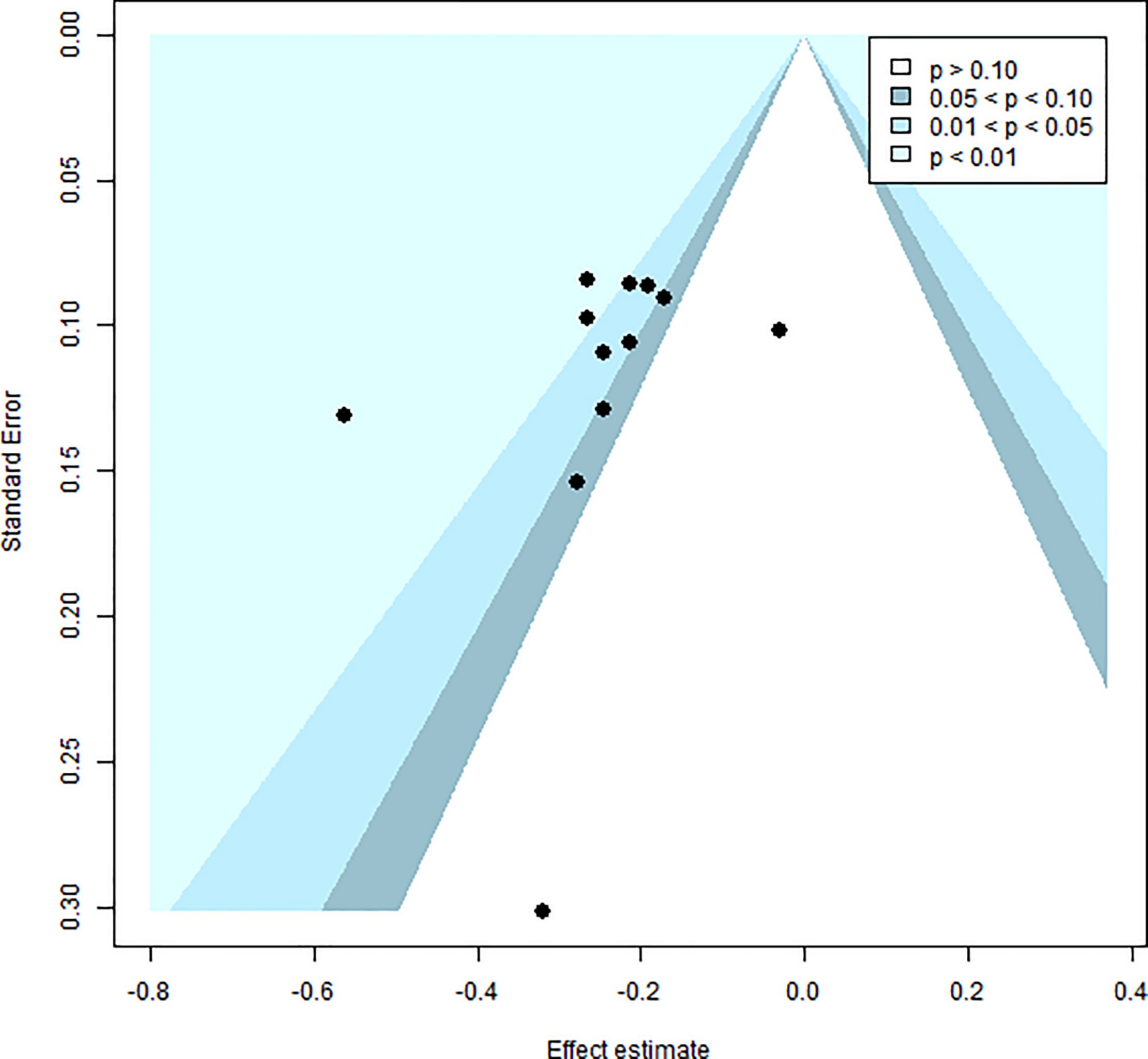
Funnel plot showing distribution of studies included in the meta-analysis of Factor 1 scores.

No significant heterogeneity was detected across studies (*Q*^*2*^ = 11.46, df = 11, p = 0.41/ *I^2^* = 4.0%). A visual inspection of the funnel plot (Fig 3) revealed that the studies were evenly distributed across varying significance levels and Egger’s regression intercept (intercept = -0.10; t = -0.82; df = 11; p = 0.43) suggested no evidence of publication bias.

### Factor 2

**Fig 4:**
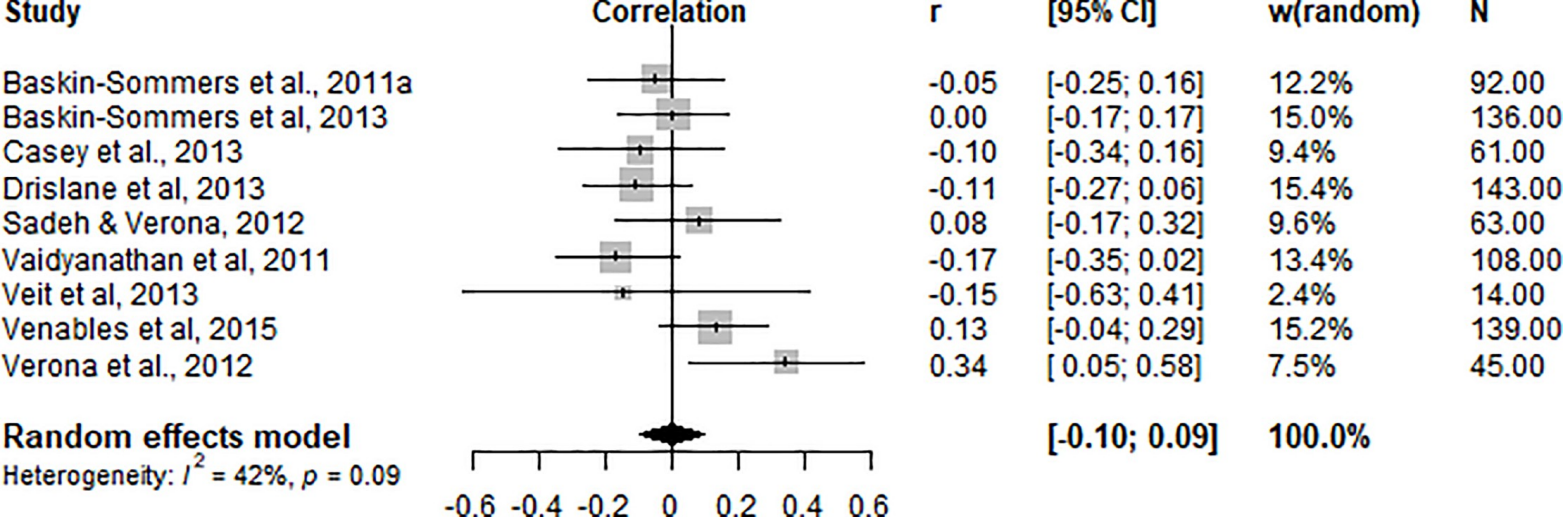
Correlations (r) between physiological threat processing index and PCL-R/SV Factor 2 scores.

Meta-analysis of nine studies indicated that Factor 2 (antisocial and impulsive/disorganised behaviours, Fig 4) scores were not significantly related to threat processing indices r = -0.005 (95% CI [-0.10, 0.09].

**Fig 5:**
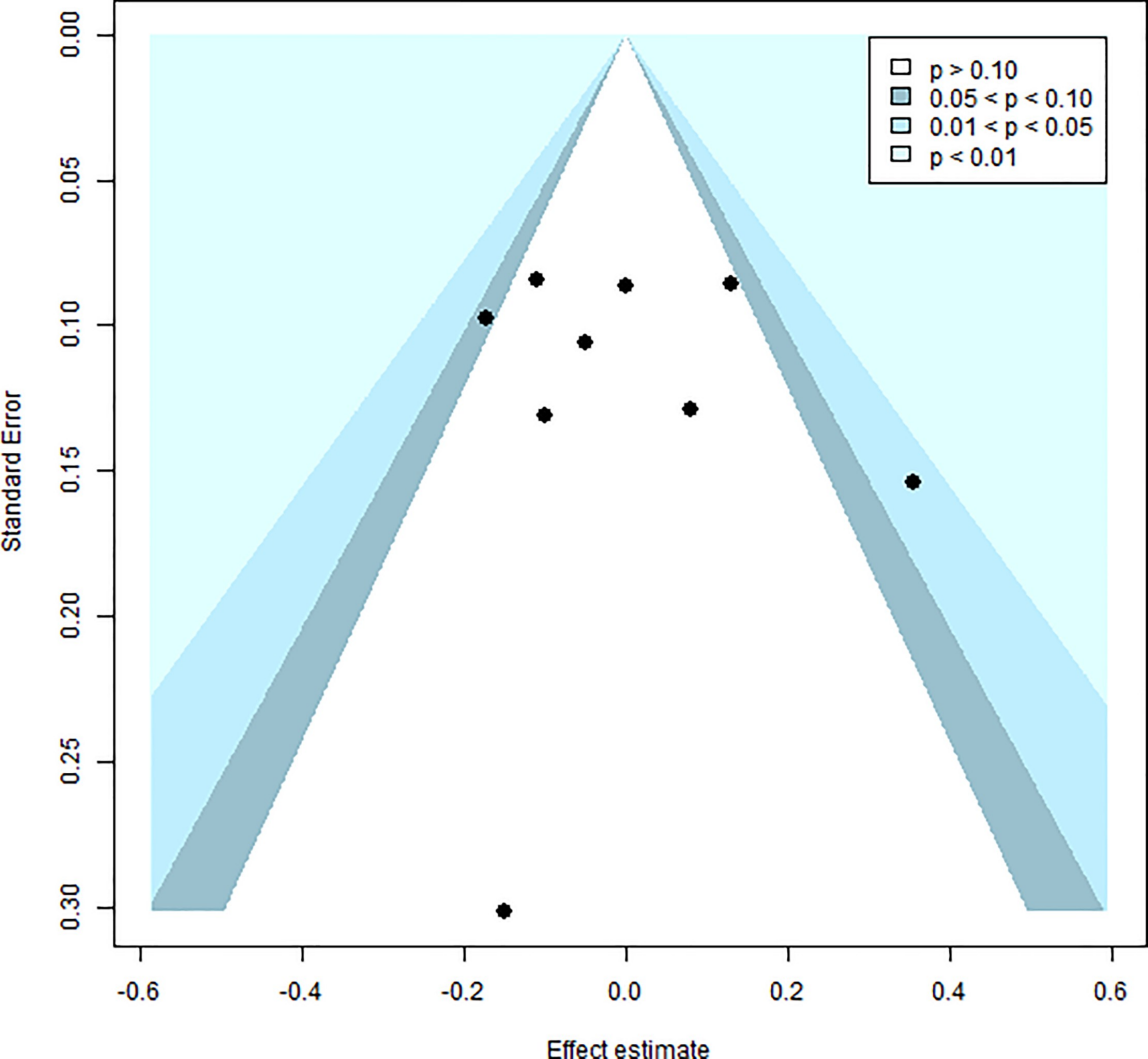
Funnel plot showing distribution of studies included in the meta-analysis of Factor 2 scores.

Heterogeneity analyses revealed no significant between-study variability (*Q*^*2*^ = 13.75, df = 8, p = 0.09/*I*^*2*^ = 41.8%). A visual inspection of the funnel plot (Fig 5) and Egger’s regression intercept (intercept = -0.07; t = -0.42; df = 8; p = 0.68) suggests that there is no publication bias.

The meta-analysis of the two separate factors did not return significant heterogeneity results, thus no meta-regression analyses were carried out.

## Discussion

This meta-analysis examined the relationship between Factor 1 and 2 scores of the psychopathy construct and physiological indices of threat processing in cross sectional studies of male offenders which reported factorial data, identifying 12 studies involving 1112 individuals for Factor 1 scores, and 9 studies involving 801 individuals for Factor 2 scores. The only previous meta-analysis in the field included data from community and student samples which utilised self-report measures [4], which rendered the potential relationship between threat processing measures such as skin-conductance [60], fear-potentiated startle [14, 63, 67] and startle blink modulation [32] and individual factor scores non-significant. For clinicians who utilise the psychopathy construct categorically to help to inform treatment programmes, this relationship required further exploration in a restricted sample of clinician-assessed offenders.

Our findings support the hypothesis that threat processing deficits in male offenders are significantly related to only one of the psychopathy factors, namely Factor 1. Meta-analytic investigation revealed that psychopathy total score and psychopathy Factor 2 scores are not associated with fear responses. Analysis of Factor 1 and threat processing revealed a significant inverse association, indicating that higher scores on this psychopathy factor are associated with greater deficits in threat processing. The effect size was significant and consistent across studies. Heterogeneity was low and not significant, further supporting the consistency of the effect direction across studies. In contrast, threat processing was not significantly related to the Factor 2 traits of psychopathy. Heterogeneity estimates here were moderate and not significant. A smaller number of studies was included in this meta-analysis, yet they consistently reported non-significant relationships between the variables of interest (see Table 1 and Fig 3).

The current findings cannot be readily integrated into the low-fear model, which argues that diminished responsivity to threat lies at the core of the condition, giving rise to other key deficits [12, 69]. Our results, in line with previous empirical investigations [34, 62, 63, 67–69] do not support the notion that impaired threat responsivity is associated with psychopathy as a unitary construct. By contrast, the dual-process model posits that aetiologically distinct pathways lead to the development of the two factors, with threat processing deficits being particularly associated with emotional detachment traits and deficient regulatory control being particularly associated with the life-span persistent antisocial features [70, 71]. Our results fit in with the larger body of empirical evidence on this model specifically linking factor 1 psychopathic traits, and not life-span persistent antisocial behaviour, to an impaired threat processing system [32, 33, 35].

A substantial number of the studies in the current meta-analyses utilised startle responses as measures of threat processing, and these reactions are presumed to be modulated via limbic systems, with a particularly important role for the amygdala [72]. The significant link between Factor 1 and threat processing impairments reported here is consistent with the view that affective deficits in psychopathy are related to atypical structure and function within affective brain systems [73–78]. The amygdala is also presumed to control the early stage processing of threatening stimuli [79] and studies utilising methods restricting conscious awareness, such as backward masking and continuous flash suppression, have shown that it is precisely the affective deficits in antisocial populations that are positively associated with impairments in early stage processing of fearful stimuli [80, 81].

### Study limitations

It should be noted that readers need to interpret the current findings in the context of restrictions inherent in our meta-analytic approach. Thus, we included those studies which examined physiological measures of threat response in male offender populations assessed with a clinician administered diagnostic tool, and where effect size measurements were included for both factors. We were unable to secure unpublished data to help to inform the meta-analysis, which may in turn have impacted on the generalizability of the findings. Nevertheless, we sought to clearly establish factor structure associations in the clinical samples with whom we work in custodial settings to help to inform our aetiological considerations and potential future approaches to treatment. Future work could employ moderation analyses to interrogate the possibility that differences may emerge when community samples on the psychopathy continuum [42–44] or female populations [47, 48] are examined.

It was beyond of the scope of the current work to investigate metrics of threat-processing beyond physiological measures. However, previous meta-analytic work on emotion recognition in psychopathy strongly supports the conclusions drawn here [39]. The global psychopathy construct was associated with pervasive deficits in recognition of emotion (fear, sadness, anger, happy, disgust), but Factor 1 scores were specifically associated with impairments in processing fear. Taken together, the literature suggests that Factor 1 is associated with deficient threat processing across different metrics.

Heterogeneity in the meta-analysis of Factor 2, albeit statistically non-significant, indicated the presence of moderate variation. Our analyses were also limited by missing data. Some of the studies identified as eligible did not report effect sizes for Factor 2 so they could not be included, although their results stated that Factor 2 was not significantly related to the outcome (see Table 1).

### Treatment implications

Traditional treatments within the criminal justice system are relatively ineffective for psychopathic offenders [82–84]. One possible explanation is that these treatments do not address the unique patterns of dysfunctions present in psychopathic individuals. Findings that the two factors are associated with distinctive cognitive-affective functions, from our studies and others [40, 85–87], strongly suggest that developing evidence-based treatments depends upon targeting the unique factor-specific deficits. Directly translating the current results into clinical practice would suggest that individuals with higher scores on Factor 1 will not be able to utilise aversive learning to shape behaviour, and so alternative strategies are required. Cognitive remediation training targeting the dysfunctions associated with the two factors have shown promising preliminary results [40].

## Conclusions

The current findings suggest that impairments in threat processing among psychopathic offenders are significantly associated with scores on Factor 1 but not Factor 2 of the psychopathy construct. These meta-analyses highlight the importance of investigating and evaluating the discrete relationships the two factorial constructs of psychopathy may have with aetiological variables. Developments in therapeutic approaches require just such a nuanced understanding.

## Supporting information

S1 Checklist PRISMA 2009 checklist. (PDF)

S1 Table Table presenting the number of excluded/included papers per database and search. (PDF)

S2 Table Table presenting the summary of the analyses reporting relevant beta and F statistics. (PDF)

S1 File Quality assessment was based on the following criteria. (PDF)

S1 Fig Correlations (r) between physiological threat processing index and PCL-R/SV Total scores. (TIF)

S2 Fig Funnel plot showing distribution of studies included in the meta-analysis of total scores. (TIF)
