# Supplementary material for: Impaired processing of threat in psychopathy: A systematic review and meta-analysis of factorial data in male offender populations
Source: PLoS One. 2019 Oct 29;14(10):e0224455. doi: 10.1371/journal.pone.0224455 (PMC6818800; doi:10.1371/journal.pone.0224455)
Supplement: S1 Table — (PDF) [file pone.0224455.s002.pdf]

S1 Table. Presenting the number of excluded/included papers per database and search.

Database		Excluded*	Not an offender sample	Not a male sample	Not a clinician administered measure	No report of physiological threat measures	Not an adult sample (over 18)	No results reported for the separate factors	Included
Initial search									
Web of Science (1077 results)		944	24	9	7	27	49	7	(Venables et al., 2015)<br><br>(Decety et al., 2013)<br><br>(Veit et al., 2013)<br><br>(Sadeh & Verona, 2012)<br><br>(Baskin-Sommers et al., 2013)<br><br>(Drislane et al., 2013)<br><br>(Baskin-Sommers et al., 2011a)

									(Vaidyanathan et al., 2011)<br><br>(Baskin-Sommers et al., 2011)<br><br>(Newman et al., 2010)
PubMed (614 results)		531	14	3	2	16	27	12	(Tillem et al., 2016)<br><br>(Venables et al., 2015)<br><br>(Baskin-Sommers et al., 2013)<br><br>(Casey et al., 2013)<br><br>(Drislane et al., 2013)<br><br>(Sadeh & Verona, 2012)<br><br>(Baskin-Sommers et al., 2011)

									(Baskin-Sommers et al., 2011a)<br><br>(Newman et al., 2010)
PsycInfo (220 results)		219					1		
Secondary search									
Web of Science (75 results)		11	13	1	2	11	30	6	(Newman et al., 2010)
PubMed (63 results)		30	7		3	5	14	3	(Newman et al., 2010)
Psycinfo (214 results)		213					1		

* Not relevant / no treat measurement/ abstract only/ review paper /same sample as an already included study/ small sample (less than 10 subjects per group)
