# Supplementary material for: Impaired processing of threat in psychopathy: A systematic review and meta-analysis of factorial data in male offender populations
Source: PLoS One. 2019 Oct 29;14(10):e0224455. doi: 10.1371/journal.pone.0224455 (PMC6818800; doi:10.1371/journal.pone.0224455)
Supplement: S2 Table — (PDF) [file pone.0224455.s003.pdf]

S2 Table. Presenting the summary of the analyses reporting relevant beta and F statistics.

Paper	Analyses	Factors/predictors included in analyses	Results
Newman et al., 2010	GLM<br><br>DV: Fear potentiated startle	Condition: threat focus, alternative low load focus, alternative high load focus<br>PCL-R Total	Significant interaction between fear potentiated startle and focus of attention (threat vs alternative focus) in psychopathic individuals.<br>Psychopathy inversely associated with fear potentiated startle in the alterative focus condition.<br>Psychopathy is not associated high load (high vs low).
		Condition: threat focus, alternative low load focus, alternative high load focus<br>PCL-R Factors	Factor 1 inversely associated with fear potentiated startle in the alterative focus condition.<br>Factor 2 not reported (authors note Factor 2 performed similarly to Factor 1 in this analysis, but there is no numerical data).
Baskin-Sommers et al., 2013	GLM<br><br>DV: startle response	Valence: pleasant, neutral, unpleasant<br>Familiarity: familiar vs novel<br>PCL-R Total score	Psychopathic individuals display deficient emotion-modulated startle for novel stimuli only.
		Valence: pleasant, neutral, unpleasant<br>Familiarity: familiar vs novel<br>PCL-R Factors	Factor 2 is associated with deficient emotion-modulated startle for novel stimuli, suggesting their deficit is more general to emotion processing (including pleasant and unpleasant stimuli).<br><br>Factor 1 specifically show a deficit for fear (unpleasant pictures), with magnitude of unpleasant vs neutral contrast decreasing as Factor 1 scores increase.
Venables et al., 2015	GLM<br><br>DV: Late positive potential	Valence: pleasant, neutral, aversive<br>PCL-R Factors	Neither psychopathy factor displayed a significant main effect.<br><br>There was a significant Factor 1 X valence interaction, lesser augmentation of LPP for aversive versus pleasant pictures with increasing levels of PCL-R Factor 1.

	(electrocortical response)		No Factor 2 X Valence interaction.
Baskin-Sommers et al., 2011a	GLM<br><br>DV: Fear potentiated startle	Focus: alternative vs threat<br>Timing of attentional cue: Early vs late<br>PCL-R Total	Psychopathy was significantly inversely related to FPS in the early alternative focus condition (high psychopathy scores are associated with decreasing FPS only when attention is engaged for an alternative task prior to presentation of threat-relevant information).
		Focus: alternative vs threat<br>Timing of attentional cue: Early vs late<br>PCL-R Factors	Both Factor 1 and Factor 2 interacted significantly with the early alternative focus condition, replicating results for total scores.<br><br>Significant main effect for Factor 1 – high factor 1 scores are associated with decreased FPS in all conditions.
Casey et al., 2013	Bivariate correlations, step-wise regression<br><br>DV: Heart rate	PCL-R Factor 1<br>Years of education<br>Risk score (HCR20)<br>PCL-R Total<br>PCL-R Factor 2	Significant predictors: Factor 1<br><br>Variance explained: 26%<br><br>For every 1 point increase in Factor 1, increased heart rate when experiencing negative pictures will be smaller by approximately half a beat per minute.
Baskin-Sommers et al., 2011b	GLM<br><br>DV: Fear potentiated startle	Focus: threat vs alternative<br>Attentional load: high vs low<br>PCL-R Total	No significant results.
		Focus: threat vs alternative<br>Attentional load: high vs low<br>PCL-R Factors	Factor 1 significantly associated with diminished FPS.
Tillem et al., 2016	GLM	Valence: pleasant, neutral, unpleasant<br>Familiarity: familiar vs novel	Lower response to unpleasant vs pleasant novel pictures; higher response to unpleasant vs pleasant familiar pictures.

	DV: theta phase coherence (EEG measure, readiness to perceive and integrate sensory inputs)	PCL-R Total<br><br><br>Valence: pleasant, neutral, unpleasant<br>Familiarity: familiar vs novel<br>PCL-R Factors	Factor 1 is associated with higher coherence during unpleasant vs pleasant familiar stimuli.<br>Factor 2 is associated with higher coherence in response to affective (both pleasant and unpleasant pictures) compared to neutral during novel trials.
