# Supplementary material for: Impaired processing of threat in psychopathy: A systematic review and meta-analysis of factorial data in male offender populations
Source: PLoS One. 2019 Oct 29;14(10):e0224455. doi: 10.1371/journal.pone.0224455 (PMC6818800; doi:10.1371/journal.pone.0224455)
Supplement: S1 File — (PDF) [file pone.0224455.s004.pdf]

S1 File. Quality assessment was based on the following criteria:

(1) Were the settings described in relation to periods and locations of recruitment, data collection duration, location?

(2) Were key elements of the study design presented early in the paper?

(3) Did the study describe eligibility (inclusion and exclusion) criteria?

(4) Was the sampling method (probability vs non-probability) representative of the population intended in the study? (with probability sampling method scoring 1)?

(5) Was a response rate stated in the study (scoring as 0 if response rate is below 60)?

(6) Were details of methods of measurement given for each variable of interest?

(7) Does the study describe any efforts to address potential sources of bias?

(8) Was a power analyses carried out?

(9) Were the statistical analyses appropriate?

(10) Did the paper discuss any confounding issues/strategies to address such issues?
