# Supplementary figures and images for: Impaired processing of threat in psychopathy: A systematic review and meta-analysis of factorial data in male offender populations
Source: PLoS One. 2019 Oct 29;14(10):e0224455. doi: 10.1371/journal.pone.0224455 (PMC6818800; doi:10.1371/journal.pone.0224455)

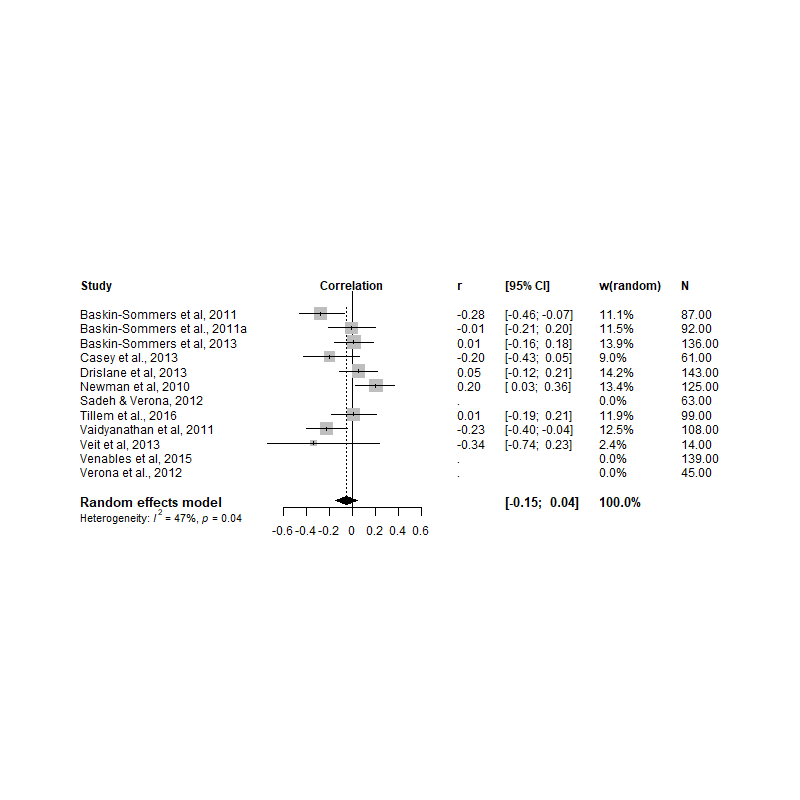

Supplement: S1 Fig — (TIF) [file pone.0224455.s005.tif]

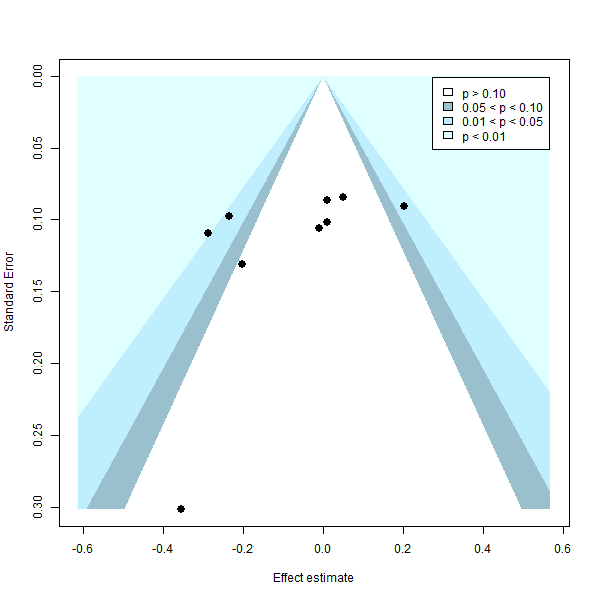

Supplement: S2 Fig — (TIF) [file pone.0224455.s006.tif]
